# A highly-active, stable and low-cost platinum-free anode catalyst based on RuNi for hydroxide exchange membrane fuel cells

**DOI:** 10.1038/s41467-020-19413-5

**Published:** 2020-11-06

**Authors:** Yanrong Xue, Lin Shi, Xuerui Liu, Jinjie Fang, Xingdong Wang, Brian P. Setzler, Wei Zhu, Yushan Yan, Zhongbin Zhuang

**Affiliations:** 1 Beijing Advanced Innovation Center for Soft Matter Science and Engineering, Beijing University of Chemical Technology, 100029 Beijing, China; 2 State Key Lab of Organic-Inorganic Composites, Beijing University of Chemical Technology, 100029 Beijing, China; 3 Department of Chemical and Biomolecular Engineering, University of Delaware, Newark, DE 19716 USA; 4 Beijing Key Laboratory of Energy Environmental Catalysis, Beijing University of Chemical Technology, 100029 Beijing, China

**Keywords:** Electrocatalysis, Fuel cells, Fuel cells

## Abstract

The development of cost-effective hydroxide exchange membrane fuel cells is limited by the lack of high-performance and low-cost anode hydrogen oxidation reaction catalysts. Here we report a Pt-free catalyst Ru_7_Ni_3_/C, which exhibits excellent hydrogen oxidation reaction activity in both rotating disk electrode and membrane electrode assembly measurements. The hydrogen oxidation reaction mass activity and specific activity of Ru_7_Ni_3_/C, as measured in rotating disk experiments, is about 21 and 25 times that of Pt/C, and 3 and 5 times that of PtRu/C, respectively. The hydroxide exchange membrane fuel cell with Ru_7_Ni_3_/C anode can deliver a high peak power density of 2.03 W cm^−2^ in H_2_/O_2_ and 1.23 W cm^−2^ in H_2_/air (CO_2_-free) at 95 °C, surpassing that using PtRu/C anode catalyst, and good durability with less than 5% voltage loss over 100 h of operation. The weakened hydrogen binding of Ru by alloying with Ni and enhanced water adsorption by the presence of surface Ni oxides lead to the high hydrogen oxidation reaction activity of Ru_7_Ni_3_/C. By using the Ru_7_Ni_3_/C catalyst, the anode cost can be reduced by 85% of the current state-of-the-art PtRu/C, making it highly promising in economical hydroxide exchange membrane fuel cells.

## Abstract (web summary)

Development of hydroxide exchange membrane fuel cells (HEMFCs) requires high-performance and low-cost catalysts for hydrogen oxidation reaction at the anode. Here the authors report Ru_7_Ni_3_/C as anode catalysts, delivering high power density and good durability in alkaline media for HEMFCs.

## Introduction

Hydrogen fuel cell is an efficient energy conversion technology that converts chemical energy directly into electricity^1,2^. However, the most popular fuel cell technology, proton exchange membrane fuel cells (PEMFCs), relies on expensive Pt-based catalysts and perfluorinated membranes^3,4^. The high cost hampered their large scale commercial application. Hydroxide exchange membrane fuel cells (HEMFCs), which are structurally similar to PEMFCs but operate in alkaline medium, have attracted much attention because of the possibility to use non-noble electrocatalysts and cheaper bipolar plates^5–8^. Currently, some highly stable hydroxide exchange membranes have been developed, making HEMFCs promising^9^. Recently, the peak power density (PPD) for H_2_/Air (CO_2_-free) HEMFCs can exceed 1 W cm^−2^ by using Pt-based catalysts^10–13^. If HEMFCs can maintain high performance without using Pt, low-cost fuel cells can be realized, thus significantly advancing the hydrogen economy.

Some Pt-free oxygen reduction reaction (ORR) catalysts (e.g. Fe–N–C, Ag, and Mn–Co oxides) have been developed in base^14–16^. They showed high activity and potential to replace the Pt-based cathode catalysts in HEMFCs. However, the hydrogen oxidation reaction (HOR) still relies on Pt-based catalysts. More importantly, the HOR/hydrogen evolution reaction (HER) activities of platinum-group metal (PGM) catalysts (e.g. Pt, Ir, and Pd) drop approximately 100-fold when changing the electrolyte from acid to base^17–19^. The relatively sluggish HOR kinetics in HEMFCs makes the anode Pt loading much higher than that in PEMFCs to achieve similar performance^5,10,17^. Therefore, the development of low-cost and high-activity anode catalysts is now required to fulfill the commercialization of HEMFCs.

Some non-platinum HOR catalysts have been reported, such as Ir- and Pd-based catalysts^20^. They showed comparable HOR activity to that of Pt/C in RDE tests, and acceptable HEMFCs performances with PPD of 200–500 mW cm^−2^ ^21–23^. Although some non-precious metal-based HOR catalysts have been reported and significant progress has been made in the past decade, their activity and stability are still lower than the precious metal-based HOR catalysts^24–27^. Therefore, the HEMFC is still in urgent need of replacement for Pt/C in anode. In this paper, we examine Ru-based HOR catalysts. Although Ru is classified as a precious metal, its price is only 6–36% of Pt in recent 5 years, having the advantages in cost. And with decent HOR activity but low ORR activity, Ru is one of the best options to mitigate the reverse current mechanism, which causes cathode carbon corrosion during start-up and shut-down^28^. Recent reports show that Ru enhances the HOR activity of PtRu/C catalyst which has the highest activity in HEMFCs^29,30^. However, Ru itself has low HOR activity. HEMFC using Ru/C as anode catalyst can only output a PPD of ca. 250 mW cm^−2^ ^31^. And because the major component of PtRu/C catalyst is Pt, it still has high cost.

Here, we report the Ru_7_Ni_3_/C catalyst as a highly active HOR catalyst, which is totally Pt-free and has improved HOR activity than the previously reported Ru-based catalyst, and the Ru_7_Ni_3_/C even has higher activity than the state-of-the-art PtRu/C catalyst, in both RDE and MEA test. The HOR mass activity and specific activity of Ru_7_Ni_3_/C is about 21 and 25 times that of Pt/C, and 3 and 5 times that of PtRu/C, respectively. The fabricated HEMFCs can deliver a high PPD of 2.03 W cm^−2^ in H_2_/O_2_ and 1.23 W cm^−2^ in H_2_/air (CO_2_-free), with the anode catalyst cost of only 15% to the state-of-the-art HEMFC with PtRu/C anode. The HEMFC with the Ru_7_Ni_3_/C anode also exhibited less than 5% degradation after 100-h cell operation at 500 mA cm^−2^ under H_2_/air (CO_2_-free). The improvement for the Ru-based HOR catalyst is attributed to the alloying effect of the additional Ni in the interior of the catalyst nanoparticles, and the enhanced water adsorption from the surface Ni oxides. The obtained Ru_7_Ni_3_/C catalyst is highly promising for efficient and durable HEMFCs in the reduction of anode catalyst cost.

## Results

### Catalyst synthesis and characterization

**Fig. 1:**
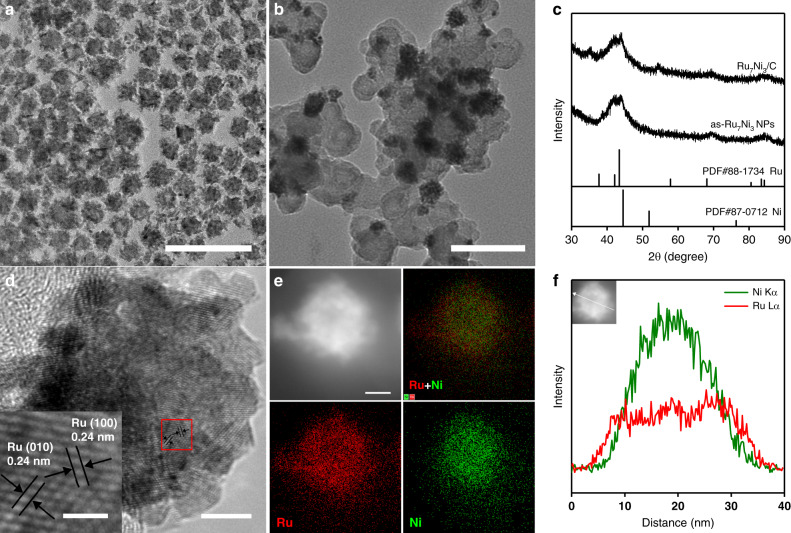
Characterizations of the Ru_7_Ni_3_/C catalysts. **a** Transmission electron microscopy (TEM) image of the as-Ru_7_Ni_3_ NPs (scale bar, 100 nm). **b** TEM image of the Ru_7_Ni_3_/C catalyst (scale bars, 100 nm). **c** The X-ray powder diffraction (XRD) patterns of the as-Ru_7_Ni_3_ NPs and Ru_7_Ni_3_/C. **d** High-resolution TEM (HRTEM) images of the Ru_7_Ni_3_/C (scale bar, 5 nm). Inset shows the corresponding red box region (scale bar, 1 nm). **e** High-angle annular dark-field (HAADF) scanning TEM (STEM) image and corresponding energy-dispersive X-ray spectrometry (EDX) mapping showing the distribution of Ru and Ni of a nanoparticle in Ru_7_Ni_3_/C (scale bar, 10 nm). **f** EDX line-scanning profile of a nanoparticle in Ru_7_Ni_3_/C. Inset shows the HAADF-STEM image of the corresponding nanoparticle.

The RuxNiy nanoparticles (NPs) were synthesized by a simple hydrothermal method. The Ru/Ni ratio of the obtained NPs can be controlled by the ratio of the Ru and Ni metal sources. Figure 1a shows the transition electron microscopy (TEM) image of the typical as-synthesized Ru_7_Ni_3_ NPs (denoted as as-Ru_7_Ni_3_ NPs, which is unsupported and uncalcined). These NPs have an average size of ca. 22 nm (Supplementary Fig. 1), and are built up by smaller NPs of ca. 5 nm. The as-Ru_7_Ni_3_ NPs were further supported on high surface area carbon, and then calcined in air at 200 °C to obtain the supported catalyst Ru_7_Ni_3_/C. During the calcination, the remaining oleylamine ligands on the surface were removed, and as-Ru_7_Ni_3_ NPs also slightly oxidized. The TEM image of Ru_7_Ni_3_/C (Fig. 1b) shows that the as-Ru_7_Ni_3_ NPs were well dispersed on the carbon supports and the NPs maintained their morphology after calcination.

The X-ray diffraction (XRD) pattern of Ru_7_Ni_3_/C (Fig. 1c) reveals the major hexagonal close-packed (hcp) Ru phase of the catalysts. The peaks are slightly shifted to higher angle, demonstrating the alloy with Ni. The high-resolution TEM (HRTEM, Fig. 1d) image of Ru_7_Ni_3_/C clearly indicates the lattice fringe with an interplanar spacing of 0.24 nm, which is consistent with the (100) plane and the (010) plane of the hcp Ru. Figure 1e shows the energy-dispersive X-ray spectrometry (EDX) elemental mapping images of a nanoparticle in Ru_7_Ni_3_/C. It demonstrates that Ru and Ni are evenly distributed in the NP. However, from the overlapped image of Ru and Ni shown in Fig. 1e, it can be found that Ni is concentrated in the interior of the NP and Ru is slightly segregated in the surface. The EDX scanning profile (Fig. 1f) further confirmed the Ru enriched surface. The Ru/Ni mass ratio in Ru_7_Ni_3_/C is ca. 75/25, tested by the inductively coupled plasma optical emission spectra (ICP-OES).

### High HOR activity of the Ru_7_Ni_3_/C measured by rotating disk electrode

**Fig. 2:**
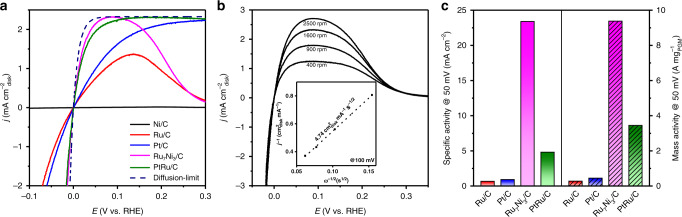
Hydrogen oxidation reaction (HOR) activity tested by the RDE method. **a** HOR polarization curves of Ru/C, Ni/C, Pt/C, PtRu/C, and Ru_7_Ni_3_/C in H_2_-saturated 0.1 M KOH solution. Scan rate: 1 mV s^−1^; rotation rate: 1600 rpm. All of the catalysts with a loading of 3.9 µg_PGM_ cm^−2^, except 3.9 µg_Ni_ cm^−2^ for Ni/C. **b** Polarization curves of Ru_7_Ni_3_/C in H_2_-saturated 0.1 M KOH at a scan rate of 1 mV s^−1^ at different rotation rates. Inset shows the Koutecky–Levich plot of Ru_7_Ni_3_/C at an overpotential of 100 mV. **c** Electrochemical surface area (ECSA) normalized specific activity and precious metal (Ru or Pt)-based mass activity at 50 mV of the Ru/C, Pt/C, PtRu/C, and Ru_7_Ni_3_/C.

The electrocatalytic performance toward HOR of the Ru_7_Ni_3_/C catalyst was firstly investigated by the rotating disk electrode (RDE) method in 0.1 M KOH electrolyte using a standard three-electrode system. The benchmark HOR catalyst, PtRu/C, and the monometallic catalyst Pt/C (commercial), Ru/C and Ni/C (synthesized, TEM images shown in Supplementary Fig. 2 and XRD patterns shown in Supplementary Fig. 3) were tested at the same condition for comparison. Figure 2a shows the HOR/HER polarization curves of these catalysts. The anode current increases sharply with increasing potential when employing Ru_7_Ni_3_/C as the catalyst, demonstrating high catalytic performance. The HOR performance of Ru_7_Ni_3_/C is higher than that of Pt/C and Ru/C, and it is even higher than that of the state-of-the-art HOR catalyst PtRu/C. Pristine Ni/C shows negligible HOR activity compared with these precious metal-containing catalysts. The current density drops when the potential is higher than 0.1 V, probably due to the surface passivation of the catalyst at high potential^32^. We also tested the polarization curves at different rotating speed (Fig. 2b). The limiting current density increases along with the elevation of the rotating speed, demonstrating the H_2_ mass-transport controlled process. The Koutecky–Levich plot is shown in the inset of Fig. 2b. A slope of 4.74 cm^2^ mA^−1^ s^−1/2^ is obtained, which is reasonably close to the theoretical number (4.87 cm^2^ mA^−1^ s^−1/2^) and indicates the current is only derived from the two-electron transfer HOR process^17^.

In order to quantitatively compare the HOR activity of the Ru_7_Ni_3_/C with other catalysts, we calculated the specific activity based on the electrochemical surface area (ECSA) and mass activity of the catalysts. The ECSAs of the catalysts were determined by copper underpotential deposition (Cu_upd_) stripping voltammetry (Supplementary Fig. 4 and Supplementary Table 1). The kinetic current density of HOR was derived by the Koutecky–Levich equation. Note that although ultra-low catalyst loadings were used in the RDE experiments to move the polarization curves away from the diffusion overpotential curve, errors may still exist due to the ultrahigh activity of the Ru_7_Ni_3_/C and PtRu/C catalysts. We also normalized the activity to the precious metal loadings on the electrode, and obtained the mass activity. The HOR specific activities and mass activities at 50 mV of the catalysts are summarized in Fig. 2c and Supplementary Table 1. The Ru_7_Ni_3_/C has an ultrahigh mass activity of 9.4 A mg_Ru_^−1^, which is 33, 21, and 3 times as high as that of Ru/C (0.28 A mg_Ru_^−1^), Pt/C (0.45 A mg_Pt_^−1^) and PtRu/C (3.5 A mg_Pt+Ru_^−1^), respectively. The specific activity of Ru_7_Ni_3_/C is 23.4 mA cm^−2^, which is 35, 25, and 5 times as high as that of Ru/C (0.67 mA cm^−2^), Pt/C (0.93 mA cm^−2^), and PtRu/C (4.8 mA cm^−2^), respectively. We also derived the exchange current (*i*_0_) of HOR/HER by fitting with the Butler–Volmer equation, and the Ru_7_Ni_3_/C catalyst also shows the highest HOR activity (Supplementary Fig. 5 and Supplementary Table 2). The relationship between the Ru/Ni ratio and HOR activity was investigated (Supplementary Figs. 6, 7 and Supplementary Table 3), and the Ru_7_Ni_3_/C is the optimized composition.

### High-performance HEMFCs by using the Ru_7_Ni_3_/C as anode catalyst

**Fig. 3:**
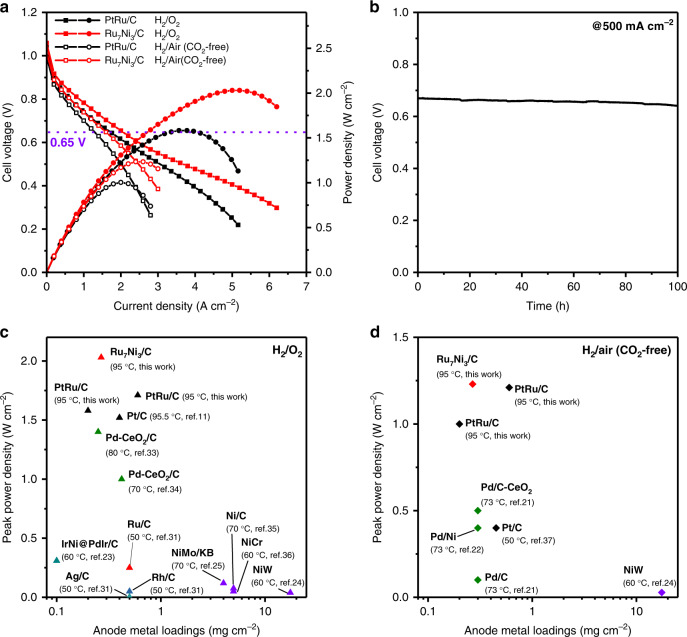
Hydroxide exchange membrane fuel cells (HEMFCs) performance and durability. **a** Polarization and power density curves of H_2_/O_2_ and H_2_/air (CO_2_-free) HEMFCs with Ru_7_Ni_3_/C (0.2 mg_Ru_ cm^−2^) or PtRu/C (0.2 mg_PtRu_ cm^−2^) in anode and Pt/C (0.4 mg_Pt_ cm^−2^) in cathode. Test conditions: cell temperature at 95 °C, anode humidifier temperature at 88 °C and cathode humidifier temperature at 97 °C, H_2_ flow rate at 1.0 L min^−1^ and O_2_/CO_2_-free air flow rate at 2.0 L min^−1^, backpressures were symmetric at 250 kPag. **b** H_2_/CO_2_-free air HEMFCs durability test at a constant current density of 0.5 A cm^−2^ with Ru_7_Ni_3_/C (0.1 mg_Ru_ cm^−2^) in anode and Pt/C (0.4 mg_Pt_ cm^−2^) in cathode. Test conditions: cell temperature at 80 °C, anode humidifier temperature at 79 °C and cathode humidifier temperature at 80 °C, H_2_ flow rate at 0.5 L min^−1^ and CO_2_-free air flow rate at 0.5 L min^−1^, backpressures were symmetric at 150 kPag. **c**, **d** The summary of the reported H_2_/O_2_ and H_2_/air (CO_2_-free) HEMFC performances using different anode catalysts measured in this paper and the Pt-free catalysts in the reported literature, respectively.

Encouraged by the high HOR activity of the obtained Ru_7_Ni_3_/C catalyst, we employed it as the anode catalyst for HEMFCs performance and durability test. A membrane electrode assembly (MEA) was fabricated by using Ru_7_Ni_3_/C (0.2 mg_Ru_ cm^−2^) as the anode, commercial Pt/C (0.4 mg_Pt_ cm^−2^) as the cathode, and PAP-TP-85 as the membrane. A similar MEA, substituting the state-of-the-art PtRu/C (0.2 mg_PtRu_ cm^−2^) at anode, was fabricated as the benchmark. Figure 3a shows the comparison for the polarization and power density curves of the HEMFCs with different anode catalysts under both H_2_/O_2_ and H_2_/air (CO_2_-free). The current density at 0.65 V cell voltage (typical operating potential for fuel cell for automotive applications) and the PPD of the Ru_7_Ni_3_/C-based HEMFCs show excellent performance that is even better than PtRu/C-based HEMFCs under both O_2_ and air (CO_2_-free). For the H_2_/O_2_ cell, the Ru_7_Ni_3_/C-based HEMFC can deliver a current density of 2.0 A cm^−2^ at the cell voltage of 0.65 V, which is higher than that of the PtRu/C-based HEMFC (1.68 A cm^−2^). The Ru_7_Ni_3_/C-based HEMFC can achieve a high PPD of 2.03 W cm^−2^ at 5.0 A cm^−2^, which is also higher than that for PtRu/C-based HEMFC (PPD of 1.58 W cm^−2^ at 3.8 A cm^−2^). Similar results can also be seen for the H_2_/air (CO_2_-free) cell. The Ru_7_Ni_3_/C-based HEMFC can deliver a current density of 1.6 A cm^−2^ at 0.65 V and PPD of 1.23 W cm^−2^ at 2.6 A cm^−2^ under air (CO_2_-free), which is one of the best H_2_/Air (CO_2_-free) HEMFC performance that has been reported and shows the advantages compared with the PtRu/C-based HEMFC (current density of 1.24 A cm^−2^ at 0.65 V and PPD of 1.0 W cm^−2^ at 2.0 A cm^−2^). To achieve a similar fuel cell performance to the Ru_7_Ni_3_/C-based HEMFCs, the anode catalyst loadings required to increase to 0.6 mg_PtRu_ cm^−2^ by using the state-of-the-art PtRu/C catalysts (polarization curves shown in Supplementary Fig. 8). The price of Ru has varied in the range of 1.6 to 9.7 $ g^−1^ in the recent 5 years, which is 6–36% to that of Pt (ca. 27.0 $ g^−1^). Coupled with the lower required loadings to achieve a similar PPD and current density at 0.65 V, the cost on the anode side can be reduced by 85% of that of the state-of-the-art HEMFCs. The used PAP-TP-85 membrane, which can afford the high operating temperature of 95 °C, is beneficial to obtain the ultrahigh HEMFC performance. We also tested the HEMFCs at 80 °C (Supplementary Fig. 9), and the Ru_7_Ni_3_/C-based H_2_/O_2_ HEMFC also shows high performance with a PPD of 1.69 W cm^−2^ at 3.86 A cm^−2^. Apart from performance, durability is another essential property for HEMFCs as it gains more and more attention recently. As can be seen in Fig. 3b, with even lower anode catalyst loading (0.1 mg_Ru_ cm^−2^ Ru_7_Ni_3_/C), the cell suffered only about 4.4% voltage loss operating at a constant current density of 0.5 A cm^−2^ under H_2_/air (CO_2_-free) after 100 h, demonstrating the good durability of the Ru_7_Ni_3_/C-based HEMFC. Supplementary Table 4 summarized the previous various Pt-free anode HEMFCs that have been published in the literature. The PPD of HEMFCs using different anode catalyst were summarized in Fig. 3c, d with their loadings^11,21–25,31–37^. An ideal catalyst should give high PPD at low catalyst loading, i.e. locates at the up-left corner in the plots. Clearly, the Ru_7_Ni_3_/C is the highly efficient anode catalyst for both H_2_-O_2_ and H_2_/air (CO_2_-free) HEMFCs. Compared with the other non-platinum catalysts, the Ru_7_Ni_3_/C shows much higher PPD.

### Surface composition of the Ru_7_Ni_3_/C catalyst

**Fig. 4:**
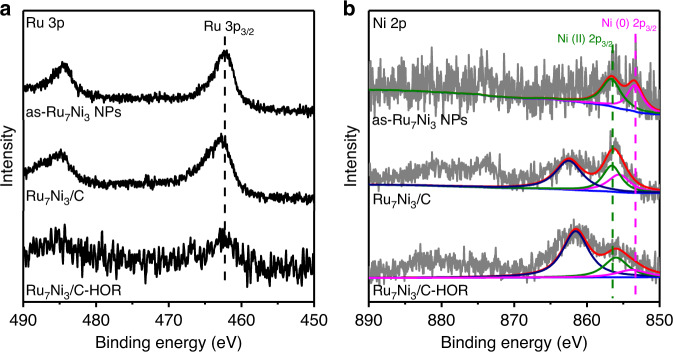
High-resolution X-ray photoelectron spectra (XPS) spectra. **a** Ru 3p. **b** Ni 2p spectra of as-Ru_7_Ni_3_ NPs, Ru_7_Ni_3_/C, and Ru_7_Ni_3_/C-HOR, respectively.

The high HOR activity of Ru_7_Ni_3_/C is attributed to its structure and composition, especially the surface composition. Based on the aforementioned characterizations, RuNi alloy was obtained. However, the surface condition may vary. X-ray photoelectron spectroscopy (XPS) analysis was employed to investigate the surface elements and their valence states. Three samples were studied, which are the as-synthesized (as-Ru_7_Ni_3_ NPs), after dispersed on carbon and calcined in air (Ru_7_Ni_3_/C) and after the HOR test (Ru_7_Ni_3_/C-HOR). Figure 4a shows the Ru core level 3p_3/2_ XPS spectra. In the calcination step, the nanoparticles were also partially oxidized, indicating by the shift of Ru peaks towards higher binding energies. After the HOR test, the Ru peaks shifted back to lower binding energies, demonstrating that Ru was reduced to metallic form because of the low test potential and the presence of the reductive H_2_ environment^38^. However, the condition of surface Ni is different. Figure 4b shows the Ni core level 2p XPS spectra. The Ni 2p_3/2_ peaks at 853 eV and 856 eV are assigned to the metallic Ni (0) and Ni (II) oxidation states, and shakeup satellite signal appears at 861 eV^39,40^. After the calcination in air, Ni was also oxidized, confirmed by the significant increase of the Ni (II) peak. After the HOR test, the Ni (II) peak still predominated, suggesting the existence of Ni oxides layer on the surface of the catalysts when doing HOR. The oxidation of Ni only occurs at the surface of the NPs, and the interior of the NPs are still RuNi alloy according to the previous studies^39^.

## Discussion

**Fig. 5:**
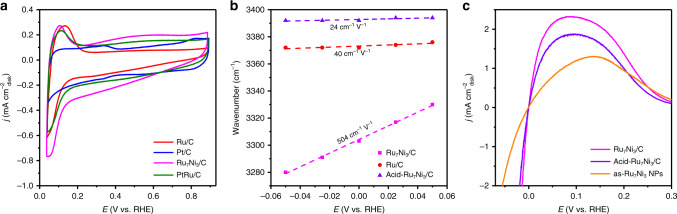
Electrochemical analyses. **a** Cyclic voltammograms of Ru/C, Pt/C, PtRu/C, and Ru_7_Ni_3_/C in Ar-saturated 0.1 M KOH at a scan rate of 50 mV s^−1^. All of the catalysts with a loading of 3.9 µg_PGM_ cm^−2^. **b** Changes of O−H stretching wavenumber of interfacial water for Ru/C, Ru_7_Ni_3_/C, and Acid-Ru_7_Ni_3_/C in H_2_-saturated 0.1 M KOH electrolyte. **c** HOR polarization curves of as-Ru_7_Ni_3_ NPs, Ru_7_Ni_3_/C, and Acid-Ru_7_Ni_3_/C catalysts in H_2_-saturated 0.1 M KOH solution. Scan rate: 1 mV s^−1^; rotation rate: 1600 rpm.

Ru is considered as the HOR active sites for the Ru_7_Ni_3_/C catalyst because of its metallic form in the HOR process. For the HOR, the H_2_ is first adsorbed on the surface of the catalysts to form adsorbed hydrogen (H*), and then electron transfer occurs so that H* is desorbed to form H^+^. Thus, the intrinsic hydrogen binding energy between H* and metal (HBE = Δ*G*^0^_H*_) is clearly critical for the HOR catalytic activity^41^. The previous studies show that water binding energy Δ*G*^0^_H2O_ is also important for HOR/HER and the Volcano relationship of apparent hydrogen binding energy (HBE_app_ = Δ*G*^0^_app_ = HBE−Δ*G*^0^_H2O_) of the catalysts to their HOR activities^42,43^. The catalyst with optimized HBE_app_, i.e. the HBE_app_ is close to 0 eV, would give the highest HOR activity. The H_upd_ peak position is related to the real HBE_app_ of the catalysts in the electrolyte^18,42^. However, for Ru-based catalyst, hydroxide may adsorb at low potential, and the peak at low potential is assigned to the exchange reaction of H* to adsorbed OH (OH*)^44,45^. From the CV curves shown in Fig. 5a, all the catalysts containing Ru show this peak at low potential of ca. 0.1 V. We can see that the peak for Ru_7_Ni_3_/C is located at the lowest potential, which means the HBE_app_ of Ru_7_Ni_3_/C is closest to zero if assuming all the Ru containing catalysts have the same influence of OH adsorption, and thus the highest HOR activity.

Zheng et al. proposed that the adsorption of H required the replacement and/or reorganization of water molecules on the surface of the catalyst^42^. Thus, the HBE_app_ is the net results of the catalyst hydrogen binding (HBE) and the water adsorption (Δ*G*^0^_H2O_). Weakened the Ru−H binding and enhanced water adsorption can both make the HBE_app_ close the optimized condition. The Ru−H binding strength was characterized by the electrochemical in situ attenuated total reflection surface-enhanced infrared absorption spectroscopy (ATR-SEIRAS). As shown in Supplementary Fig. 10, the Ru−H bands shifted from 2094 cm^−1^ for Ru/C to 2082 cm^−1^ for Ru_7_Ni_3_/C. The lower wavenumber corresponds to a weaker HBE, indicating the alloying with Ni can weaken the Ru−H interactions^46^. Previous studies show that the HBE can be tuned by making alloy. For example, the DFT-calculated Δ*G*_H*_ on Pt (111) plane (−0.33 eV) is lower than that of Pt_3_Ru(111) plane (−0.19 eV), because of the weakening in the Pt−H* interaction by the electronic effect of Ru alloying^30^. Similarly, the additional Ni can also weaken Ru−H interactions.

The enhanced water adsorption on the Ru_7_Ni_3_/C was also observed by the in situ ATR-SEIRAS. Supplementary Fig. 10 shows the obtained IR spectra recorded during stepping the potential from 0.2 to −0.15 V vs. RHE. The broad peak located at around 3400 cm^−1^ was assigned to the O−H stretching vibration of the interfacial water^47^. A larger Stark tuning rate of 504 cm^−1^ V^−1^ was found for Ru_7_Ni_3_/C than that of Ru/C (40 cm^−1^ V^−1^), demonstrating the stronger interaction of water molecules with the Ru_7_Ni_3_/C catalysts (Fig. 5b)^48,49^. The enhanced water adsorption on the Ru_7_Ni_3_/C was attributed to the surface Ni oxides. The metal oxides are more hydrophilic than its corresponding metals (Supplementary Table 5). As a result, the surface Ni oxides could promote the adsorption of water.

To confirm the critical role of the surface oxidized Ni species on the HOR, we conducted a series of control experiments. We compared the HOR activity of the as-Ru_7_Ni_3_ NPs and the calcined Ru_7_Ni_3_/C. An enhanced HOR was observed for the calcined samples, implying the significance of the surface Ni oxides (Fig. 5c). Note, the as-Ru_7_Ni_3_ NPs are covered by oleylamine, which is used in the synthesis. Thus, its HOR activity is underestimated. To further confirm the function of surface Ni oxides, we washed the Ru_7_Ni_3_/C in 0.5 M H_2_SO_4_ for 12 h (named as Acid-Ru_7_Ni_3_/C) to remove the surface Ni oxides (TEM image shown in Supplementary Fig. 11). The XPS result for the Acid-Ru_7_Ni_3_/C confirms that most of the surface Ni was removed (Supplementary Fig. 12). Acid-Ru_7_Ni_3_/C was found to have lower HOR activity than Ru_7_Ni_3_/C (Fig. 5c), further demonstrating the critical role of the surface Ni oxides. The in situ ATR-SEIRAS results show that the interfacial water has a weaker binding when the surface Ni oxides were washed out, indicating by the lower Stark tuning rate of O−H stretching vibration for Acid-Ru_7_Ni_3_/C (Fig. 5b). However, the Ru−H bands remained almost unchanged after acid wash (Supplementary Fig. 10, 2084 cm^−1^ for Acid-Ru_7_Ni_3_/C compared with 2082 cm^−1^ for Ru_7_Ni_3_/C). These results demonstrate that the enhanced water adsorption is mainly contributed by the surface Ni oxides, and the interior Ni weakened the Ru−H interaction. Both effects contributed to the optimization of HBE_app_, and lead to the high HOR activity of Ru_7_Ni_3_/C.

In summary, a Pt-free Ru_7_Ni_3_/C HOR catalyst with high performance has been successfully synthesized. It shows a mass activity 33, 21, and 3 times that of Ru/C, Pt/C and PtRu/C in the RDE test, respectively. The HEMFCs using Ru_7_Ni_3_/C as anode catalyst also show high peak power density of 2.03 mW cm^−2^ in H_2_/O_2_ and 1.23 mW cm^−2^ in H_2_/air (CO_2_-free), which are better than the HEMFCs using PtRu/C. The catalyst also demonstrates excellent stability in the HEMFC durability test, with less than 5% voltage decay after 100 h at 500 mA cm^−2^ under H_2_/air (CO_2_-free). The high performance of the Ru_7_Ni_3_/C catalyst is originated from the weakened HBE_app_, which is attributed to the alloying effect of Ni to Ru, and the enhanced water adsorption from the surface oxidized Ni species. This Ru_7_Ni_3_/C is highly promising in the HEMFCs because of its high HOR activity, good stability, and significantly lower cost than PtRu/C.

## Methods

### Synthesis of the RuNi/C catalyst

For a typical synthesis of as-Ru_7_Ni_3_ NPs, 0.056 mmol of ruthenium(III) 2,4-pentanedionate and 0.024 mmol of nickel(II) 2,4-pentanedionate were dissolved in a mixed solution of oleylamine (6 mL) and toluene (2 mL) and then ultrasonicated for 30 min. After that, 0.216 mmol of 4-dimethylaminobenzaldehyde was added to the solution and stirred for another 30 min. Next, it was transferred into a 10 mL Teflon-lined stainless-steel autoclave and heated to 220 °C and maintained at this temperature for 12 h. Then, the NPs were collected by centrifugation at 11,000 rpm for 5 min and washed three times with absolute ethanol.

The obtained as-Ru_7_Ni_3_ NPs were redispersed in cyclohexane, and 16.5 mg of carbon (Vulcan XC-72) was added. The mixture was suction filtered and collected, then it was calcined in air at 200 °C for 5 h using a tube oven to obtain the Ru_7_Ni_3_/C catalyst.

### Physical characterization

The X-ray powder diffraction (XRD) patterns were recorded on a Rigaku D/Max 2500 VB2 + /PC X-ray powder diffractometer equipped with Cu Kα radiation (λ = 0.154 nm). Transmission electron microscopy (TEM) images were obtained with a JEOL JEM-1230 transmission electron microscope operating at 100 kV. High-resolution TEM (HRTEM) and energy-dispersive X-ray spectrometry (EDX) elemental mapping were performed using an FEI Tecnai G2 F30 Super-Twin high-resolution transmission electron microscope at an accelerating voltage of 300 kV. The X-ray photoelectron spectra (XPS) were measured using a Thermo Fisher ESCALAB 250Xi XPS system with a monochromatic Al Kα X-ray source. All binding energies were calibrated to the C 1 s peak (284.8 eV). Inductively coupled plasma optical emission spectroscopy (ICP-OES, Optima 7300 DV, Perkin Elmer) was used to determine the metal contents of as-prepared catalysts.

### Electrochemical tests by RDE

Electrochemical measurements were performed in a standard three-electrode system controlled by a potentiostat (V4, Princeton Applied Research). A catalyst-coated glassy carbon electrode (5 mm in diameter), a graphite rod, and a saturated calomel electrode (SCE) used as the working, counter and reference electrode, respectively. The catalyst ink was prepared by dispersing the catalysts in water/ethanol (1:3, v/v) with 0.05 wt% Nafion, then the ink was drop cast on the GC electrode to form a catalyst thin film. The platinum-group-metal (Ru and/or Pt) loading on the electrode was controlled as 3.9 μg_PGM_ cm^−2^, which was determined by the inductively coupled plasma optical emission spectrometer (ICP-OES, Optima 7300 DV, Perkin Elmer). The recorded potentials were referred to RHE with iR-correction. The zero point of RHE was determined by the equilibrium potential of HER/HOR using Pt/C as working electrode rotating at 1600 rpm in H_2_-saturated electrolyte. The solution resistance (*R*) was measured using AC-impendence spectroscopy from 200 kHz to 100 mHz with a voltage perturbation of 10 mV. Cu_upd_ stripping voltammetry (0.276–0.675 V, 10 mV s^−1^) was performed in an Ar-purged 0.5 M H_2_SO_4_ solution containing 5 mM of CuSO_4_ after Cu deposition at 0.276 V for 100 s. The voltammogram on each catalyst in 0.5 M H_2_SO_4_ solution without CuSO_4_ (0.025–0.675 V, 10 mV s^−1^) was applied as the background for the corresponding Cu_upd_ stripping voltammogram.

### In situ ATR-SEIRAS experiments

The attenuated total reflectance surface-enhanced infrared absorption spectroscopy (ATR-SEIRAS) experiments were taken with Nicolet iS50 FT-IR spectrometer equipped with an MCT detector cooled with liquid nitrogen and PIKE VeeMAX III variable angle ATR sampling accessory. The spectral resolution was set to 8 cm^−1^ and 64 interferograms were co-added for each spectrum. The spectra are given in absorption units defined as *A* = − log(*R*/*R*_0_), where *R* and *R*_0_ represent the reflected IR intensities corresponding to the sample- and reference-single beam spectrum, respectively.

A 60° Si face-angled crystal was used as reflection element. The angle of incidence was set as ca. 70°. The ultra-thin Au film was deposited chemically in it for IR-signal enhancement and conduction of electrons. The electrocatalyst was dropped onto Au film to serve as a working electrode for SEIRAS experiments with the loading of 0.05 mg cm^−2^. A platinum wire and an SCE electrode were used as counter and reference electrode in all tests, respectively. The H_2_-saturated 0.1 M KOH was used as the electrolyte. Chronopotentiometry method was used in this experiment at different potentials (−0.15 to 0.2 V vs. RHE without iR-correction). The SEIRAS spectra were collected during the chronopotentiometry test. And the reference-single beam spectrum is collected at 0.5 V.

### MEA tests

The synthesized Ru_7_Ni_3_/C or the PtRu/C (14 wt% Pt and 6 wt% Ru on Vulkan XC-72, Sainergy) were used as the anode catalyst. Pt/C (HiSpec 4000, 40 wt% Pt, Alfa Aesar) was used as the cathode catalyst with the loading of 0.4 mg_Pt_ cm^−2^. The hydroxide exchange membrane and ionomer (PAP-TP-*x*, *x* is the molar ratio between *N*-methyl-4-piperidone and terphenyl monomers) were synthesized as reported before^9^. The catalyst ink was prepared by ultrasonically dispersing the catalysts and ionomer (PAP-TP-100, 3.5 wt% in ethanol for performance test and PAP-TP-85, 5 wt% in ethanol for durability test) into water and isopropanol (1:20 v/v) for 1 hour. Then the catalyst ink was sprayed onto both sides of PAP-TP-85 membrane (18 µm for performance test and 13 µm for durability test) to fabricate a catalyst-coated membrane (CCM) with the electrode area of 5 cm^−2^. All CCMs were immersed into 3 M KOH solution for 2 h (exchange the solution every 1 h) for performance test or 1 M NaHCO_3_ solution for 1 h (exchange the solution every 30 min) for durability test and then rinsed thoroughly with deionized water to remove all excess KOH or NaHCO_3_. The rinsed CCM was assembled with a fluorinated ethylene propylene (FEP) gasket, a GDL (SGL 29 BC), a graphite bipolar plate with 5 cm^2^ flow field (ElectroChem) and a metal current collector for each side to complete the full HEMFC. Fuel cell test system (Scribner 850e) equipped with a backpressure module was used for all the HEMFC tests.

## Supplementary information

Supplementary Information

## Data Availability

The data that support the findings of this study are available from the corresponding authors upon reasonable request.
