# Supplementary material for: A highly-active, stable and low-cost platinum-free anode catalyst based on RuNi for hydroxide exchange membrane fuel cells
Source: Nat Commun. 2020 Nov 6;11:5651. doi: 10.1038/s41467-020-19413-5 (PMC7648055; doi:10.1038/s41467-020-19413-5)
Supplement: Supplementary file 1 — Supplementary Information [file 41467_2020_19413_MOESM1_ESM.pdf]

A Highly-active, Stable and Low-cost Platinum-free Anode Catalyst Based on RuNi for Hydroxide Exchange Membrane Fuel Cells

Yanrong Xue[1,2,†], Lin Shi[3,†], Xuerui Liu[1,2], Jinjie Fang[1,2], Xingdong Wang[2], Brian P. Setzler[3], Wei Zhu[2], Yushan Yan[1,3,*] and Zhongbin Zhuang[1,2,4*]

Supplementary Information

Supplementary Note 1: Calculation of the HOR/HER exchange current density.

The kinetic current (i_k) was calculated using the Koutecky-Levich equation:

$$\frac{1}{i} = \frac{1}{i_k} + \frac{1}{i_d} \qquad \text{Supplementary Equation (1)}$$

where i is measured current and i_d is diffusion-limited current, which is obtained by Nernstian diffusional overpotential (η_d), assuming infinitely reaction kinetics[1]:

$$\eta_d = -\frac{RT}{2F}\ln(1 - \frac{i_d}{i_l}) \qquad \text{Supplementary Equation (2)}$$

where R is the universal gas constant, T is the temperature in Kelvin, F is Faraday's constant, i_l is the HOR limiting current.

The HOR/HER exchange current (i_0) was obtained by fitting the HOR/ HER kinetic current (i_k) to the Butler-Volmer equation:[1]

$$i_k = i_0(e^{\alpha_a F\eta/RT} - e^{-\alpha_c F\eta/RT}) \qquad \text{Supplementary Equation (3)}$$

Where η is the overpotential, F is the Faraday's constant, R is the universal gas constant, T is the temperature in Kelvin, α_a and α_c are the anodic and cathodic transfer coefficients, respectively. If the HOR/HER reaction pathway follows Tafel and Volmer steps, $\alpha_a + \alpha_c = 1$. On the contrary, $\alpha_a + \alpha_c = 2$ when the HOR/HER reaction pathway follows Heyrovsky and Volmer steps.[2]

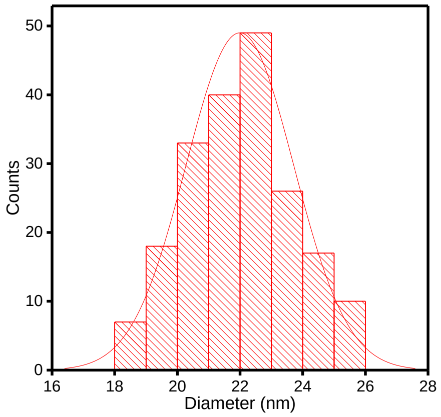

Supplementary Figure 1. The particle size distribution histogram of as-Ru_7Ni_3 NPs.

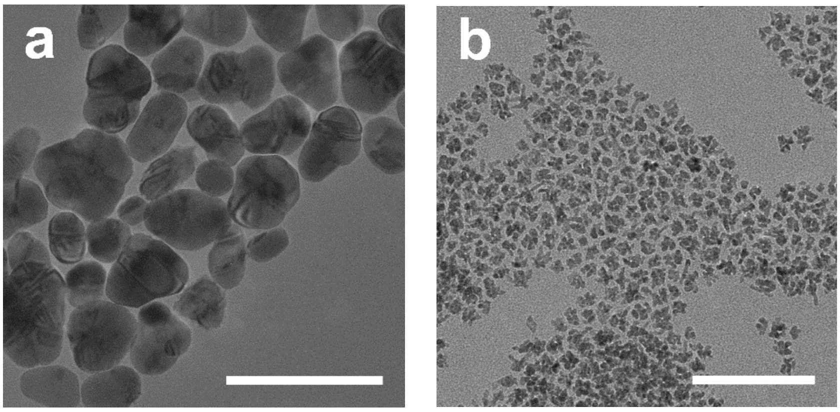

Supplementary Figure 2. TEM images of the synthesized **a**, Ni NPs (scale bar, 100 nm) and **b**, Ru NPs (scale bar, 50 nm).

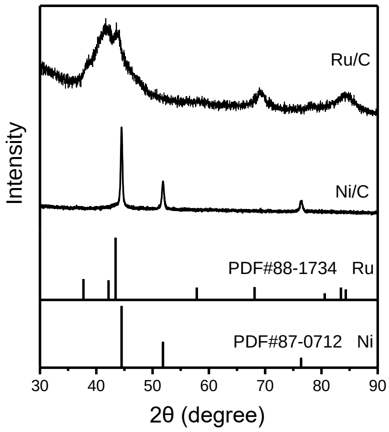

Supplementary Figure 3. XRD patterns of Ni/C and Ru/C.

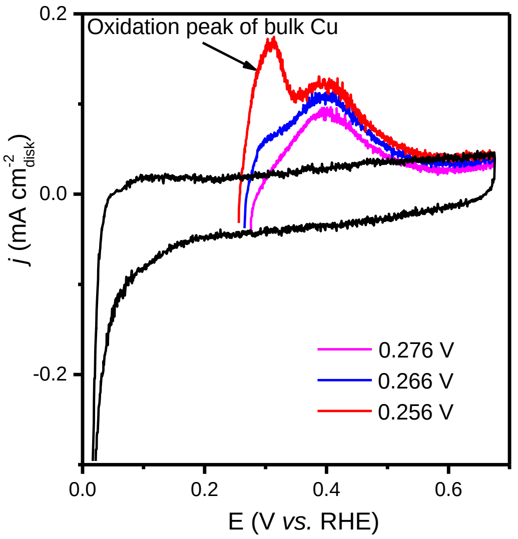

Supplementary Figure 4. The Cu_{upd} stripping voltammogram in 0.5 M H_2SO_4 with 5 mM of $CuSO_4$ on Ru_7Ni_3/C. The black curve was obtained in 0.5 M H_2SO_4 without $CuSO_4$. The electrode was firstly polarized at 0.256 V, 0.266 V and 0.276 V for 100 s to form the upd layers, respectively. Then the deposited Cu was oxidate between the underpotentially deposited potential to 0.675 V at a sweep rate of 10 mV s^{-1}. Under the polarization potentials of 0.276 V, there is only one oxidation peak around 0.4 V, which belongs to the underpotentially deposited monolayer Cu. When the potential was decreased to 0.266 V and 0.256 V, another oxidation peak appeared, which can be attributed to the oxidation of bulk copper.

Supplementary Table 1. The specific activities and mass activities at 50 mV for Ru/C, Pt/C, Ru_7Ni_3/C and PtRu/C.

Catalyst	*mass* (μg_{PGM})	*ECSA* ($m^2\ g^{-1}$)	$i_{k,\ 50\ mV}$ (mA)	$j_{s,\ 50\ mV}$ ($mA\ cm^{-2}$)	$j_{m,\ 50\ mV}$ ($A\ mg_{PGM}^{-1}$)
Ru/C	0.76	43	0.22	0.67	0.28
Pt/C	0.76	48	0.34	0.93	0.45
Ru_7Ni_3/C	0.76	40	7.1	23.4	9.4
PtRu/C	0.76	72	2.6	4.8	3.5

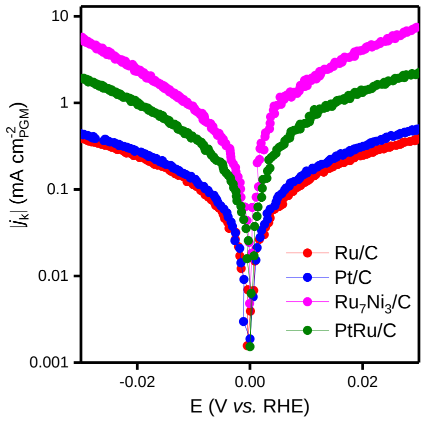

Supplementary Figure 5. Tafel plots of the HER/HOR kinetic current density for Ru/C, Pt/C, Ru_7Ni_3/C and PtRu/C.

Supplementary Table 2. The exchange current (i_0) of Ru/C, Pt/C, Ru_7Ni_3/C and PtRu/C (catalyst loadings are 0.76 μg_{PGM}).

Catalyst	$i_{0,1}$ (mA)[a]	α_1 [a]	$i_{0,2}$ (mA)[a]	α_2 [a]
Ru/C	0.09	0.47	0.04	0.99
Pt/C	0.14	0.53	0.06	1.02
Ru_7Ni_3/C	1.09	0.9	0.52	1.4
PtRu/C	0.82	0.62	0.37	1.11

[a]: $i_{0,1}$ and α_1 were obtained by assuming $\alpha_a + \alpha_c = 1$. $i_{0,2}$ and α_2 were obtained by assuming $\alpha_a + \alpha_c = 2$.

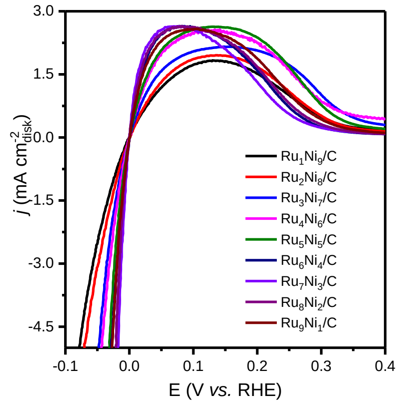

Supplementary Figure 6. HOR polarization curves of Ru_xNi_y/C catalysts in H_2-saturated 0.1 M KOH solution. Scan rate: 10 mV s^{-1}; rotation rate: 1600 rpm. All of the catalysts with a loading of 7.7 μg_{Ru} cm^{-2}.

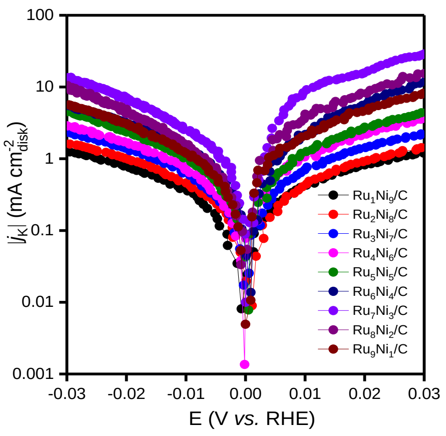

Supplementary Figure 7. Tafel plots of the HER/HOR kinetic current density for the Ru_xNi_y/C catalysts.

Supplementary Table 3. The exchange current (i_0) of the Ru_xNi_y/C catalysts (catalyst loadings are 1.51 μg_{Ru}).

Catalyst	$i_{0,1}$ (mA)[a]	α_1 [a]	$i_{0,2}$ (mA)[a]	α_2 [a]
Ru_1Ni_9/C	0.20	0.47	0.06	0.97
Ru_2Ni_8/C	0.24	0.46	0.08	0.97
Ru_3Ni_7/C	0.39	0.42	0.13	0.93
Ru_4Ni_6/C	0.60	0.57	0.20	1.07
Ru_5Ni_5/C	0.72	0.46	0.30	0.97
Ru_6Ni_4/C	1.3	0.84	0.50	1.36
Ru_7Ni_3/C[b]	1.8	1.09	0.96	1.86
Ru_8Ni_2/C	1.4	0.96	0.65	1.44
Ru_9Ni_1/C	1.1	0.60	0.45	1.10

[a]: $i_{0,1}$ and α_1 were obtained by assuming $\alpha_a + \alpha_c = 1$. $i_{0,2}$ and α_2 were obtained by assuming $\alpha_a + \alpha_c = 2$.

[b]: The difference of Ru_7Ni_3/C in here and in Table S2 comes from the different catalyst loadings.

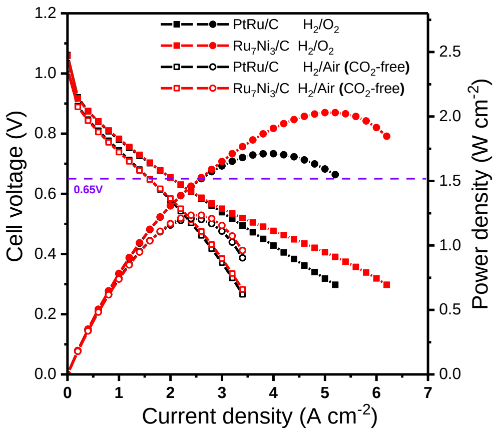

Supplementary Figure 8. Polarization and power density curves of H_2/O_2 and H_2/air (CO_2-free) HEMFCs with Ru_7Ni_3/C (0.2 mg_{Ru} cm^{-2}) or PtRu/C (0.6 mg_{PtRu} cm^{-2}, HiSpec 10000, 40 wt% Pt and 20 wt% Ru on Vulkan XC-72, Alfa Aesar) in anode and Pt/C (0.4 mg_{Pt} cm^{-2}) in cathode. Test conditions: cell temperature at 95 °C, anode humidifier temperature at 88 °C and cathode humidifier temperature at 97 °C, H_2 flow rate at 1.0 L min^{-1} and O_2/CO_2-free air flow rate at 2.0 L min^{-1}, backpressures were symmetric at 250 kPag.

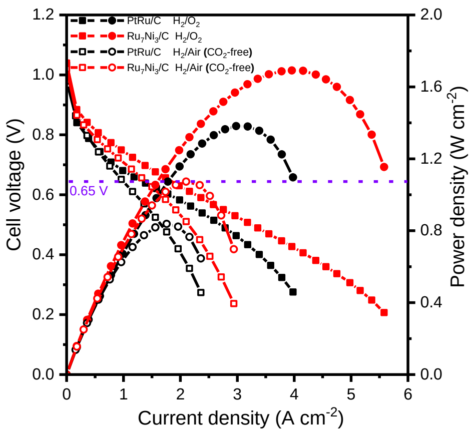

Supplementary Figure 9. HEMFCs performance and durability. (a) Polarization and power density curves of H_2/O_2 HEMFCs with Ru_7Ni_3/C (0.2 mg_{Ru} cm^{-2}) or PtRu/C (0.2 mg_{PtRu} cm^{-2}) in anode and Pt/C (0.4 mg_{Pt} cm^{-2}) in cathode. Test conditions: cell temperature at 80 °C, anode humidifier temperature at 80 °C and cathode humidifier temperature at 80 °C, H_2 flow rate at 1.0 L min^{-1} and O_2/CO_2-free air flow rate at 2.0 L min^{-1}, backpressures were symmetric at 200 kPag. For the H_2/O_2 cell, the Ru_7Ni_3/C-based HEMFC can deliver a current density of 1.74 A cm^{-2} at the cell voltage of 0.65 V, which is higher than that of the PtRu/C-based HEMFC (1.26 A cm^{-2} at the cell voltage of 0.65V). The Ru_7Ni_3/C-based HEMFC can achieve a high PPD of 1.69 W cm^{-2} at 3.86 A cm^{-2}, which is higher than that for PtRu/C-based HEMFC (PPD of 1.38 W cm^{-2} at 3.08 A cm^{-2}). The Ru_7Ni_3/C-based HEMFC can deliver a current density of 1.32 A cm^{-2} at 0.65 V and PPD of 1.07 W cm^{-2} at 2.1 A cm^{-2} under air (CO_2-free), which shows the advantages compared with the PtRu/C-based HEMFC (current density of 0.96 A cm^{-2} at 0.65 V and PPD of 0.84 W cm^{-2} at 1.76 A cm^{-2}).

Supplementary Table 4. Summary of the HEMFC performances.

	Catalyst	Catalyst loading (mg cm^{-2})	Membrane	T_{Cell} (℃)	Back pressure (kPag)	PPD for H_2/O_2 (W cm^{-2})	PPD for H_2/Air (W cm^{-2})	Ref.
anode	Ru_7Ni_3/C	0.2[a]	PAP-TP-85	95	250	2.03	1.23	This work
cathode	Pt/C	0.4		80	200	1.69	1.07	
anode	PtRu/C	0.2	PAP-TP-85	95	250	1.58	1.0	This work
cathode	Pt/C	0.4		80	200	1.38	0.84	
anode	Pt/C	0.4	PAP-TP-85	95.5	150	1.52		[3]
cathode	Pt/C	0.4			250			
anode	Pt/C	0.45	Acta S.p.A.'s Membrane	50	0		0.4	[4]
cathode	Pt/C	0.45						
anode	Pd-CeO_2/C	0.25	ETFE-BTMA Membrane	80	0	1.4		[5]
cathode	Pt/C	0.4						
anode	Pd-CeO_2/C	0.42[b]	ETFE-BTMA Membrane	70	200	1		[6]
cathode	PdCu/C	0.58[b]			100			
anode	IrNi@PdIr/C	0.1	A201 Tokuyama	60	0	0.31		[7]
cathode	Pt/C	0.3						
anode	Pd/C-CeO_2	0.3	CellEra's Membrane	73	300		0.5	[8]
cathode	Ag/C	3.0			100			
anode	Pd/C	0.3	CellEra's Membrane	73	300		0.1	[8]
cathode	Ag/C	3.0			100			
anode	Pd/Ni	0.3	CellEra's Membrane	73	300		0.4	[9]
cathode	Ag/C	3.0			100			
anode	Ru/C	0.5	A201 Tokuyama	50	0	0.25		[10]
cathode	Pt/C	0.5						
anode	Rh/C	0.5	A201 Tokuyama	50	0	0.05		[10]
cathode	Pt/C	0.5						
anode	Ag/C	0.5	A201 Tokuyama	50	0	0.002		[10]
cathode	Pt/C	0.5						
anode	Ni/C	5.0	TPQPOH152 membrane	70	250	0.076		[11]
cathode	Ag/C	0.5						
anode	NiCr	5.0	QAPS membrane	60	130	0.05		[12]
cathode	Ag/C	1.0						
anode	NiW	17.5	xQAPS	60	0	0.04	0.028	[13]
cathode	CoPPY/C	2.0						
anode	NiMo/KB	4.0	A201 Tokuyama	70	138	0.12		[14]
cathode	Pd/C	0.2						

[a]: The Ru based loading is 0.2 mg_{Ru} cm^{-2}, and the metal based loading is 0.26 mg_{metal} cm^{-2}.

[b]: These are Pd based loadings.

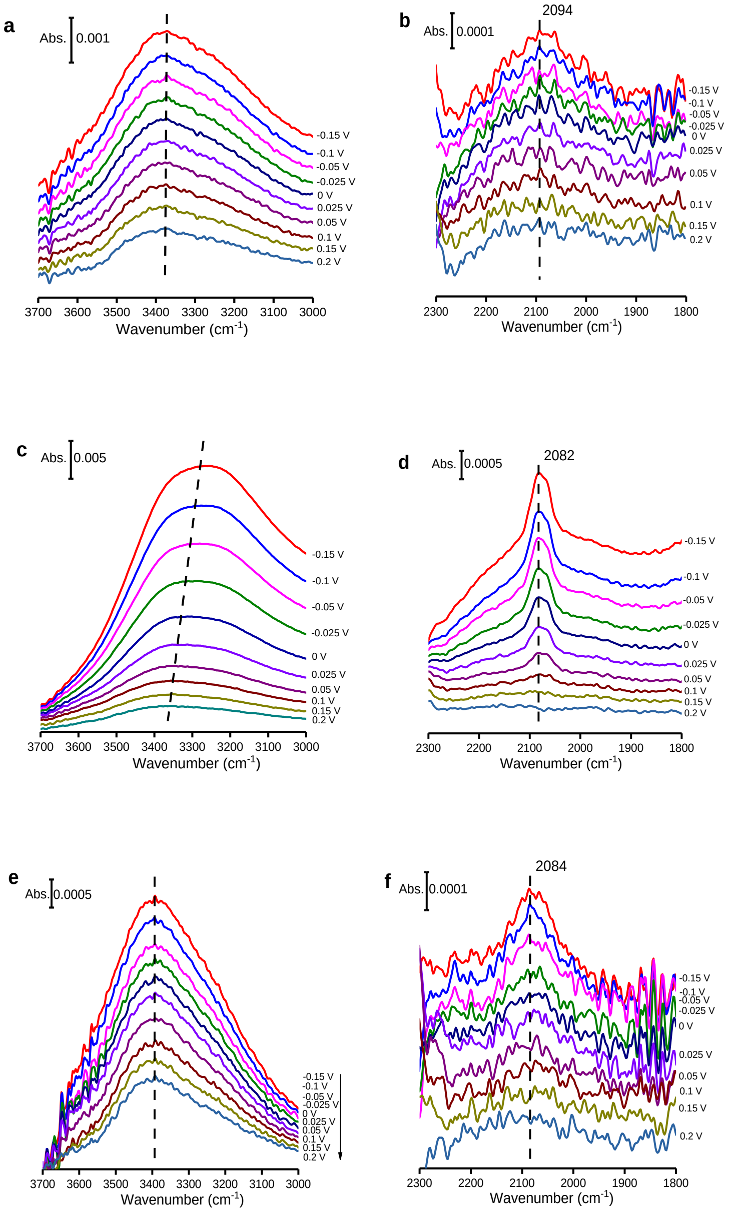

Supplementary Figure 10. *In-situ* ATR-SEIRAS spectra recorded during stepping the potential from 0.2 to −0.15 V *vs.* RHE in H_2-saturated 0.1 M KOH electrolyte for **a-b** Ru/C, **c-d** Ru_7Ni_3/C and **e-f** Acid-Ru_7Ni_3/C.

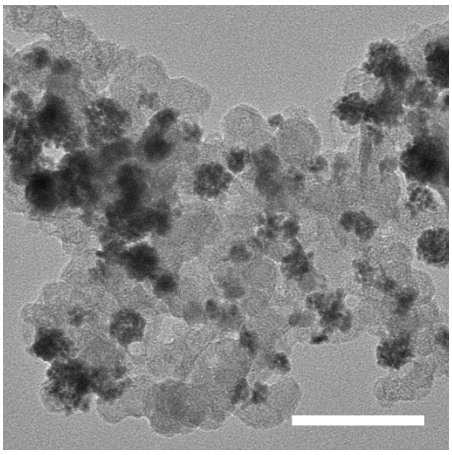

Supplementary Figure 11. TEM image of the Acid-Ru_7Ni_3/C (scale bar, 100 nm).

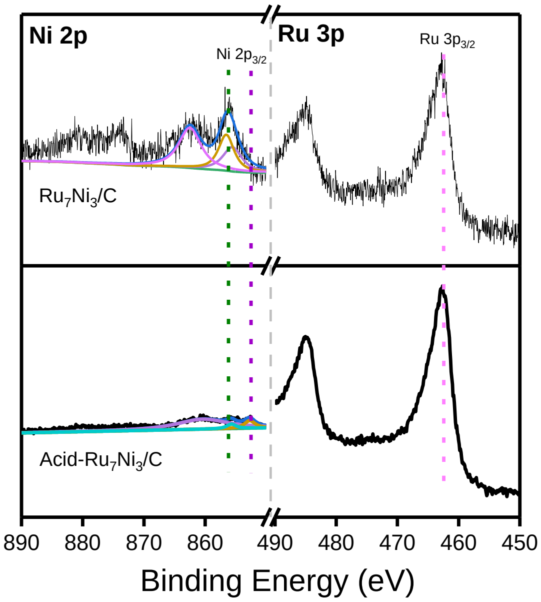

Supplementary Figure 12. High-resolution XPS spectra of Ru_7Ni_3/C and Acid-Ru_7Ni_3/C for Ni 2p and Ru 3p. The Ru:Ni ratio increased from 4:3 to 20:1 after acid wash, indicating most of Ni was washed off from the surface of the particles.

Supplementary Table 5. Adsorption energies (E_{ad}) of water on Ru(0001), Ni(100) and NiO(100) surfaces, respectively.

Surface	E_{ad} (eV)	Ref.
Ru(0001)	0.38	[15]
Ni(100)	−0.27	[16]
NiO(100)	−1.05	[17]

Supplementary References

1 Sheng, W., Gasteiger, H.A., & Shao-Horn, Y. Hydrogen oxidation and evolution reaction kinetics on platinum: Acid vs alkaline electrolytes. *J. Electrochem. Soc.* **157**, B1529-B1536 (2010).

2 Rheinlander, P.J., Herranz, J., Durst, J., & Gasteiger, H.A. Kinetics of the hydrogen oxidation/evolution reaction on polycrystalline platinum in alkaline electrolyte reaction order with respect to hydrogen pressure. *J. Electrochem. Soc.* **161**, F1448-F1457 (2014).

3 Teng W., L.S., Junhua W., Yun Z., Brian P. S., Santiago R-C., & Yushan Y. High-performance hydroxide exchange membrane fuel cells through optimization of relative humidity, backpressure and catalyst selection. *J. Electrochem. Soc.* **166**, F3305-F3310 (2019).

4 Piana, M., Boccia, M., Filpi, A., Flammia, E., Miller, H.A., Orsini, M., Salusti, F., Santiccioli, S., Ciardelli, F., & Pucci, A. H_2/air alkaline membrane fuel cell performance and durability, using novel ionomer and non-platinum group metal cathode catalyst. *J. Power Sources* **195**, 5875-5881 (2010).

5 Bellini, M., Pagliaro, M. V., Lenarda, A., Fornasiero, P., Marelli, M., Evangelisti, C., Innocenti, M., Jia, Q., Mukerjee, S., Jankovic, J., Wang, L., Varcoe, J. R., Krishnamurthy, C. B., Grinberg, I., Davydova, E., Dekel, D. R., Miller, H. A., Vizza, F. Palladium–ceria catalysts with enhanced alkaline hydrogen oxidation activity for anion exchange membrane fuel cells. *ACS App. Energy Mater.* **2**, 4999-5008 (2019).

6 Travis J. O., Xiong P., Hamish A. M., Francesco V., Lianqin W., John R. V., Dario R. D., & William E. M. Beyond 1.0 W cm^{-2} performance without platinum: The beginning of a new era in anion exchange membrane fuel cells. *J. Electrochem. Soc.* **165**, J3039-J3044 (2018).

7 Qin, B., Yu, H., Jia, J., Jun, C., Gao, X., Yao, D., Sun, X., Song, W., Yi, B., & Shao, Z. A novel IrNi@PdIr/C core-shell electrocatalyst with enhanced activity and durability for the hydrogen oxidation reaction in alkaline anion exchange membrane fuel cells. *Nanoscale* **10**, 4872-4881 (2018).

8 Hamish A. M., Alessandro L., Francesco V., Marcello M., Francesco Di B., Francesco D'A., Yair P., Miles P., & Dario R. D. A Pd/C-CeO_2 anode catalyst for high-performance platinum-free anion exchange membrane fuel cells. *Angew. Chem. Int. Ed.* **55**, 6004-6007 (2016).

9 Alesker, M., Page, M., Shviro, M., Paska, Y., Gershinsky, G., Dekel, D.R., & Zitoun, D. Palladium/nickel bifunctional electrocatalyst for hydrogen oxidation reaction in alkaline membrane fuel cell. *J. Power Sources* **304**, 332-339 (2016).

10 Ohyama, J., Sato, T., & Satsuma, A. High performance of Ru nanoparticles supported on carbon for anode electrocatalyst of alkaline anion exchange membrane fuel cell. *J. Power Sources* **225**, 311-315 (2013).

11 Gu, S., Sheng, W., Cai, R., Alia, S.M., Song, S., Jensen, K.O., & Yan, Y. An efficient Ag-ionomer interface for hydroxide exchange membrane fuel cells. *Chem. Commun.* **49**, 131-133 (2013).

12 Lu, S., Pan, J., Huang, A., Zhuang, L., & Lu, J. Alkaline polymer electrolyte fuel cells completely free from noble metal catalysts. *Proc. Natl. Acad. Sci.* **105**, 20611-20614 (2008).

13 Hu, Q., Li, G., Pan, J., Tan, L., Lu, J., & Zhuang, L. Alkaline polymer electrolyte fuel cell with Ni-based anode and Co-based cathode. *Int. J. Hydrogen Energy* **38**, 16264-16268 (2013).

14 Kabir, S., Lemire, K., Artyushkova, K., Roy, A., Odgaard, M., Schlueter, D., Oshchepkov, A., Bonnefont, A., Savinova, E., Sabarirajan, D.C., *et al.* Platinum group metal-free NiMo hydrogen

oxidation catalysts: High performance and durability in alkaline exchange membrane fuel cells. *J. Mater. Chem. A* **5**, 24433-24443 (2017).

15 Michaelides, A., Alavi, A., & King, D.A. Different surface chemistries of water on Ru{0001}: From monomer adsorption to partially dissociated bilayers. *J. Am. Chem. Soc.* **125**, 2746-2755 (2003).

16 Mohsenzadeh, A., Bolton, K., & Richards, T. DFT study of the adsorption and dissociation of water on Ni(111), Ni(110) and Ni(100) surfaces. *Surf. Sci.* **627**, 1-10 (2014).

17 Yu, N., Zhang, W.-B., Wang, N., Wang, Y.-F. & Tang, B.-Y. Water adsorption on a NiO(100) surface: A GGA+U study. *J. Phys. Chem. C* **112**, 452-457 (2008).
